# Somatotopy in the Human Somatosensory System

**DOI:** 10.3389/fnhum.2018.00235

**Published:** 2018-06-12

**Authors:** Rosa M. Sanchez Panchuelo, Julien Besle, Denis Schluppeck, Miles Humberstone, Susan Francis

**Affiliations:** ^1^Sir Peter Mansfield Imaging Centre, School of Physics and Astronomy, University of Nottingham, Nottingham, United Kingdom; ^2^Department of Psychology, American University of Beirut, Beirut, Lebanon; ^3^School of Psychology, University of Nottingham, Nottingham, United Kingdom; ^4^Nottingham University Hospitals Trust, University of Nottingham, Nottingham, United Kingdom

**Keywords:** primary somatosensory cortex, secondary somatosensory cortex, somatotopy, digits, fMRI, ultra-high field

## Abstract

Previous functional magnetic resonance imaging (fMRI) studies have demonstrated digit somatotopy in primary somatosensory cortex (SI), and even shown that at high spatial resolution it is possible to resolve within-digit somatotopy. However, fMRI studies have failed to resolve the spatial organisation of digit representations in secondary somatosensory cortex (SII). One of the major limitations of high spatial resolution fMRI studies of the somatosensory system has been the long acquisition time needed to acquire slices spanning both SI and SII. Here, we exploit the increased blood oxygenation level dependent contrast of ultra-high-field (7 Tesla) fMRI and the use of multiband imaging to study the topographic organisation in SI and SII with high spatial resolution at the individual subject level. A total of *n* = 6 subjects underwent vibrotactile stimulation of their face, hand digits and foot (body imaging) and their individual hand digits (digit mapping) for each left and right sides of the body. In addition, *n* = 2 subjects participated only in the body imaging experiment on both their left and right sides. We show an orderly representation of the face, hand digits and foot in contralateral primary cortex in each individual subject. In SII, there is clear separation of the body areas of the face, hand and foot but the spatial organisation varies across individual subjects. However, separate representation of the individual digits of the hand in SII could not be resolved, even at the spatial resolution of 1.5 mm due to largely overlapping representations.

## Introduction

With the advent of ultra-high-field (UHF) MR scanners operating at 7 Tesla (7T) and above, high spatial resolution functional Magnetic Resonance Imaging (fMRI) has been used to study the somatotopic representation of individual digits of the hand in the posterior bank of the central sulcus, corresponding to the location of primary somatosensory cortex (SI), in individual human subjects (Sánchez-Panchuelo et al., 2010; Stringer et al., 2011; Besle et al., 2014; Martuzzi et al., 2014; Kolasinski et al., 2016). UHF fMRI has also been shown to provide the spatial resolution required to resolve within-digit somatotopy, demonstrating the subdivision of cytoarchitectonic areas of SI based on functional criteria (Sánchez-Panchuelo et al., 2012, 2014) and to resolve activations of the leg and foot in Brodmann area subdivisions of SI (Akselrod et al., 2017). In contrast to SI, the spatial organisation and function of human secondary somatosensory cortex (SII), located in the parietal operculum (OP) on the upper bank of the lateral sulcus, has not been explored in humans in detail. As a result, there are only few human fMRI studies that have investigated somatotopy in SII.

Previous research in non-human primates has suggested that SII contains two distinct areas with mirror representations of the body surface, which share a common border at the representations of the face, hands, and feet (Burton et al., 1995; Krubitzer et al., 1995). Disbrow et al. (2000) proposed an analogous somatotopy in humans based on two complete fMRI body maps of the hand, foot, face, hip, and shoulder following pneumatic stimulation. Eickhoff et al. (2006b,c) have shown that the human OP can be subdivided into four cytoarchitectonic areas that are possible human homologues of subdivisions identified in non-human primates: OP1 corresponding to SII, OP2 to an inferior parietal vestibular area (PIVC), OP3 to a ventral somatosensory area (VS) and OP 4 to a parietal ventral area (PV). A subsequent fMRI study (Eickhoff et al., 2007) correlated the functional somatotopic maps with cytoarchitectonic areas OP1 and OP4, and concluded that these two areas constitute the human homologous of SII and PV, respectively, with OP3 the most possible homologue to VS.

Although the somatotopy in SII is less well defined than in SI, some fMRI studies have confirmed the gross face–hand–foot somatotopic organisation in SII (Disbrow et al., 2000; Ruben et al., 2001; Eickhoff et al., 2007) while another could not discriminate between clusters of activation in response to hand and foot stimulation in SII (Young et al., 2004). However, the finer somatotopy of individual fingers in SII remains to be resolved (Ruben et al., 2001). To the best of our knowledge, no study to date has exploited the increased blood oxygenation level dependent (BOLD) sensitivity of 7T to perform high spatial resolution fMRI of SII in an attempt to resolve digit somatotopy in this area.

Here, we apply multiband (MB) EPI, also termed simultaneous multi-slice (SMS) EPI, to achieve simultaneous high spatial resolution coverage of SI and SII at 7T whilst participants received vibrotactile stimulation either to individual digits or the hand, foot and face. This allows us to address whether the improved spatial resolution at UHF fMRI allows the representation of the body and individual digits to be resolved in SII, while simultaneously measuring the known somatotopic maps in SI.

## Materials and Methods

Magnetic resonance imaging data were collected on a 7T Philips Achieva scanner (Best, Netherlands) using a Nova Medical head volume transmit coil and a 32-channel receive coil (Wilmington, MA, United States). Experimental procedures were approved by the University of Nottingham Medical School Ethics Committee, and subjects provided written informed consent prior to the experiments.

Somatotopic mapping of the individual digits of the hand, face and foot was performed using ‘travelling wave’ (TW) paradigms (Sánchez-Panchuelo et al., 2010; Besle et al., 2013). For this study, six subjects participated in two fMRI scanning sessions. In one session, a ‘body mapping’ TW paradigm was performed to map regions of the cortex corresponding to tactile stimulation of the face, digits 2, 3, and 4, and the foot on the left side (Subjects M1, M2, M3, and M4) or right side (Subjects M1, M2, M5, and M6) of the body. In a second session, a ‘digit mapping’ TW paradigm was performed to generate a somatotopic map of the five fingertips (same side of stimulation as the first session). Two additional subjects (M7, M8) participated only in the body mapping session for both left and right side. **Table 1** shows a summary of the subjects participating in each experiment. Four additional subjects (Subjects A1, A2, A3, and A4) participated in a single fMRI scan session in which vibrotactile stimulation was independently delivered to the fingertips of the left hand in order to estimate overlap between the representation of the different digits (Independent experiment). In this experiment subjects were asked to attend to the different digits in separate blocks, but this aspect of the data will not be reported here. All subjects were right handed and had an average age of 27 ± 4 (six female and six male) for the body mapping data sets and 30 ± 4 (one female and three male) for the independent stimulation design experiment.

**Table 1:** Summary of subjects participating in each experiment.

	Left body mapping	Left digits mapping	Right body mapping	Right digits mapping
Subject M1	x	x	x	x
Subject M2	x	x	x	x
Subject M3	x	x		
Subject M4	x	x		
Subject M5			x	x
Subject M6			x	x
Subject M7	x		x	
Subject M8	x		x	


### Stimuli and Paradigm

In each scan session, vibrotactile stimulation was delivered to the skin using independently controlled piezoelectric devices which each stimulated a ∼1 mm^2^ skin area (Dancer Design^1^). Vibrotactile stimulation comprised bursts of 0.4 s duration (frequency 30 Hz) separated by 0.1 s gaps to limit habituation effects.

#### Body Mapping Experiment Using a Travelling Wave

**FIGURE 1:**
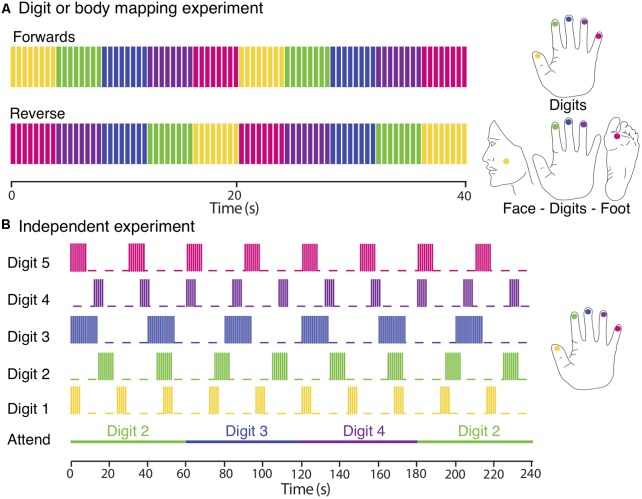
Experimental designs. **(A)** Digit and body somatotopy: data were collected using digits and face–digits–foot travelling wave paradigms (shown here for the right side of the body). Each location was sequentially stimulated for 4 s (intermittent simulation at 30 Hz; 0.4 s bursts every 0.5 s). Functional scans consisted of 10 (digit mapping experiment) or 12 (body mapping experiment) cycles and were repeated four to six times, alternating between forward and reverse order. **(B)** Independent paradigm. Each digit was stimulated with a different periodicity (yellow, Digit 1; green, Digit 2; blue, Digit 3; magenta, Digit 4; and crimson, Digit 5). Each stimulation period was formed of blocks of 0.4 s burst of continuous vibrotactile 30-Hz stimulation every 0.5 s. Attention is shifted to a different digit each 60 s. Each fMRI run lasted for 480 s and was repeated three times so that each digit was stimulated with each of the three different periodicities and each attention condition was repeated a total of eight times.

A TW paradigm was used to sequentially stimulate a location in the face under the zygomatic bone (cheek bone), the distal phalanges of Digit 2 (D2), Digit 3 (D3), Digit 4 (D4) and the sole of the foot below the hallux, on the right or left side of the body, see **Figure 1A** for the approximate locations. Subjects rested their hand, digits and foot on the piezoelectric stimulators, the face stimulator was taped to the face and subjects also held this in place with their non-stimulated hand during the fMRI experiment. Each location was stimulated for 4 s over a 20 s cycle either in a forward (Face–D2–D3–D4–Foot) or reverse (Foot–D4–D3–D2–Face) order. Each functional scan comprise of 10–12 cycles, and 4–6 functional scans were acquired, alternating between stimulation in the forward and reverse order.

#### Digit Mapping Experiment Using a Travelling Wave

A TW paradigm was used to create a travelling wave of activity between the cortical representation of the individual digits of either the right or left hand. Each of the five fingertips was sequentially stimulated from Digit 1 to Digit 5 (forward order) or from Digit 5 to Digit 1 (reverse order). Timing of the stimuli was identical to that used for the body mapping TW experiment. A total of six functional scans were acquired, alternating between stimulation in the forward and reverse order.

#### Digit Mapping Experiment Using Independent Stimulation

In this ‘independent stimulation digit mapping experiment’, vibrotactile stimulation was applied to all digits of the left hand in parallel using “on–off” boxcar waveforms of different periodicity for each digit (5 s on/19 s off, 8 s on/22 s off, or 14 s on/26 s off ±180° phase lag), see **Figure 1B**. At any point in time, stimuli were delivered to combinations of zero, one, two, or three digits. Note, that we never stimulated more than three digits at once. To control for attentional effects, subjects performed a demanding task comprising a visual display indicating to the subject to attend to either Digit 2, 3, or 4, with attention shifting to the next digit every 60 s (i.e., with a periodicity different from that of any of the digit stimulation boxcar waveforms). Each run was 480 s in duration, and was repeated three times for each subject. This ensured that each digit was stimulated with an integer number of cycles at each of the three periodicities (and stimulation lengths), resulting in a balanced number of stimulations of different length across all five-digit stimulation conditions (D1, D2, D3, D4, and D5) and a balanced number of attention trials (*n* = 8) for each of the three attention conditions (Attend 2, Attend 3, and Attend 4). Subjects were asked to count the total number of stimulation bursts to the attended digit and to report these numbers at the end of each run. Given that multiple digits were simultaneously stimulated during the experiment, participants found this to be a highly demanding task. In contrast to the travelling wave paradigm, this design allows the analysis of spatial overlap in cortical activation due to the stimulation of different digits. A detailed analysis of the effects of spatial attention on the BOLD response in the different conditions is beyond the scope of this paper.

### Imaging Protocol

fMRI data were acquired using a multiband (GyroTools Ltd., Zurich, Switzerland), single-shot gradient echo–echo planar imaging (GE–EPI) sequence with repetition time (TR) 2 s, echo time (TE) 25 ms, flip angle (FA) 75°, field of view of 192 mm × 192 mm in the anterior–posterior (A–P), right–left (R–L) directions. A SENSE acceleration factor of 2.5 was used in the A–P direction, and multi-band factor of 2 to acquire 52 slices of 1.5 mm isotropic resolution spanning the whole brain. Functional runs were followed by the acquisition of a high-resolution, T_2_^∗^-weighted axial FLASH image with the same slice prescription and coverage (0.5 mm × 0.5 mm × 1.5 mm resolution; TE/TR = 9.3/458 ms, FA = 32°, SENSE-factor = 2) to allow subsequent registration to a structural T_1_-weighted reference volume for surface rendering. For each participant, a 1 mm isotropic structural whole-head T_1_-weighted reference volume was acquired using a ‘phase sensitive inversion recovery’ (PSIR) sequence (Mougin et al., 2016) with linear k-space phase encoding scheme, TE/TR = 3.7/12 ms, FA = 8°, inversion times of 778 and 2,480 ms, and tailored RF TR-FOCI (Hurley et al., 2010) inversion pulse. This structural data was used to create visualisations of functional maps in flattened cortical patches of the somatosensory cortices.

### Data Analysis

Functional imaging data were analysed using mrTools^2^^,^^3^ in Matlab (The Mathworks; Natick, MA, United States). fMRI data sets were realigned to the last volume of the data set acquired closest in time to the high-resolution T_2_^∗^-weighted dataset (reference EPI frame). To account for scanner drift and other low-frequency signals, all time-series were high-pass filtered (0.01 Hz cut-off) and converted to percent-signal change for subsequent statistical analysis.

For each of the travelling wave ‘digit mapping experiment’ and ‘body mapping experiment’, the time series of the forward and reverse scans were combined to cancel the haemodynamic delay in the respective averaged time series (for details, see Besle et al., 2013). We then used standard Fourier-based analysis on the corrected average time series for each experiment to obtain the coherence and the phase of the best-fitting 1/20 Hz sine wave at each voxel. The phase represents the lag with respect to the onset of the stimulus, and hence indicates which digit (digit mapping experiment) or body location: face, digit or foot (body mapping experiment) was stimulated. Statistical maps were rendered onto flattened representations of both the central and post-central gyrus (SI) and the lateral sulcus in the OP (SII) of each subject’s contralateral cortex, and results were combined across subjects after non-rigid spherical normalisation (see Section ‘Surface Rendering’ and ‘Surfaced-Based Group Analysis’). For visualisation, all individual phase maps are displayed at a coherence > 0.35, with the exception of the digit maps in the SII patch shown in **Figure 5**, where the threshold was lowered to a coherence > 0.3 to reveal the maps. Former fMRI studies in visual (Freeman et al., 2011) and somatosensory (e.g., Overduin and Servos, 2008) domain have used this thresholding method.

#### Digit Mapping: Independent Stimulation

Data from all three fMRI runs of the ‘independent stimulation experiment’ were first concatenated into a single fMRI time series. Somatotopic maps of the digits in contralateral SI and SII were then formed by voxel-wise fitting of the time series with a general linear model (GLM). This allowed identification of cortical areas responding to the stimulation of each digit including all attend and non-attend stimulation trials (i.e., ignoring attention effects). In the GLM analysis, each digit stimulation sequence was modelled as a separate regressor. For each regressor, the stimulation (of different duration for each digit) was modelled as a boxcar convolved with a canonical double-gamma HRF and its orthogonalised temporal derivative, resulting in a design matrix with 10 regressors (two regressors per digit stimulation condition). The obtained parameter estimate (beta weight) for each digit stimulation condition hence represent the average of the attend and non-attend conditions. For each digit condition, we tested on a voxel-by-voxel basis whether the magnitude parameter estimate was greater than zero. False-discovery rate (FDR) adjustment (Benjamini and Hochberg, 1995) was performed using an adaptive step-up method (Benjamini et al., 2006). All adjusted *P*-values were converted to quantiles of the standard normal distribution (*Z*-score). Statistical maps for each stimulation condition were then thresholded (*Z* > 2.3, equivalent to adjusted *P* < 0.01) and transformed into flattened representations for each participant’s cortex (see below) for display purposes and ROI definition. In SI, digit-specific ROIs were defined for each of the digit stimulation conditions as contiguous cluster of voxels within the posterior-wall of the central sulcus and the precentral sulcus which survived the threshold (*P* < 0.01, FDR-corrected). In SII, a single ROI was defined as the union of the active area defined by all stimulation conditions (given that the representations of different digits largely overlap in SII, see Section ‘Results’). All ROIs were restricted to the cortical surface. These ROIs were interrogated for parameter estimates of the first GLM regressor for each digit (equivalent to the magnitude of the BOLD modulation).

#### Surface Rendering

To project the statistical maps onto flattened reconstructions of the cortical surface, statistical maps were moved from functional data space into the subject’s whole-head anatomical T_1_-weighted space in two steps: (1) align reference EPI frame to in-plane T_2_^∗^-weighted anatomical volume using non-linear alignment to account for any residual distortions in the functional volume, and (2) linearly align the in-plane T_2_^∗^-weighted anatomical volume with the reference T_1_-weighted volume. All alignment steps were performed using an iterative, multi-resolution robust estimation method (Nestares and Heeger, 2000) as implemented in mrTools.

Cortical segmentations were obtained from the whole head, T_1_-weighted anatomical volume using Freesurfer v5.3.0^4^ (Fischl et al., 1999). Flattened representations of the cortical regions surrounding the central and post-central gyrus (SI) and the lateral sulcus in the OP (SII) were obtained using the mrFlatMesh algorithm (VISTA software^5^). Statistical maps were transformed to flattened space using linear interpolation and displayed at a cortical depth half-way between the white-matter and pial surfaces.

#### Surface-Based Group Analysis

Freesurfer was used to reconstruct the cortical surface of the MNI single-subject template brain^6^ (Holmes et al., 1998). The individual left- and right-hemisphere surfaces (derived from the whole-head T_1_-weighted structural reference) were then aligned to the respective Colin27’s template surface using Freesurfer’s spherical surface-based non-rigid registration (Fischl et al., 1999).

To create group average maps, the individual right/left spherical surface meshes (Freesurfer outputs) were resampled to match the vertices of the right/left spherical surface mesh of the Colin27 template brain. Travelling-wave maps (coherence, amplitude, and phase) were projected to the Colin27 flat cortical patch, and data were then averaged across all six subjects at each coordinate. Group phase maps were formed by averaging the results from the Fourier analysis (in the complex domain, taking into account phase and amplitude) across subjects. Probability maps for the different stimulation conditions were then formed as follows: individual coherence maps were converted to *p*-values and used to threshold the phase maps (*p* ≤ 0.05, FDR-corrected). Individual activation masks for the face, D2, D3, D4, and foot were formed by dividing the threshold phase map into five equally spaced bins. An additional hand digits mask was formed by considering phase values across the three middle bins. Probability maps were then formed by adding the activation masks for each specific stimulation condition across subjects. For each stimulation condition, the probability map provides the number of subjects which shows activation at a given location (vertex) on the surface. All displayed group maps were lightly smoothed (by a Gaussian Kernel with a FWHM of 1.5 mm) in surface space and averaged across all cortical depths. ROIs for each specific stimulation condition were formed from both the group phase maps and the probability maps. The group phase map was binned as described above to provide ROIs for the face, hand digits and foot. Independent ROIs were also formed from the probability maps for each stimulation condition by considering continuous clusters of vertices whose probability > 2 (more than two subjects activating a location). A final ROI was formed by the union of both ‘phase’ and ‘probability’ ROIs for each stimulation condition. Functional data were visually compared with probabilistic maps of the OP (OP1, OP2, OP3, OP4) based on the Jülich histological atlas (Eickhoff et al., 2006a,b), which were projected from the MNI single-subject volumetric space into the flattened lateral sulcus patch.

To assess on intersubject variability, we computed the cortical distance (in surface space) between clusters from the group map (phase) and the corresponding clusters for each individual subject for face, hand and foot stimulation conditions. For every subject’s (and phase group map) clusters we determined the coordinates of the voxel with the highest coherence value and used these coordinates to compute the Euclidean distances in the 2D flattened SII patch of the template brain between the group and each individual subject. In addition, we computed the distance between each pair of clusters in the group phase maps.

## Results

### Body Somatotopy

**FIGURE 2:**
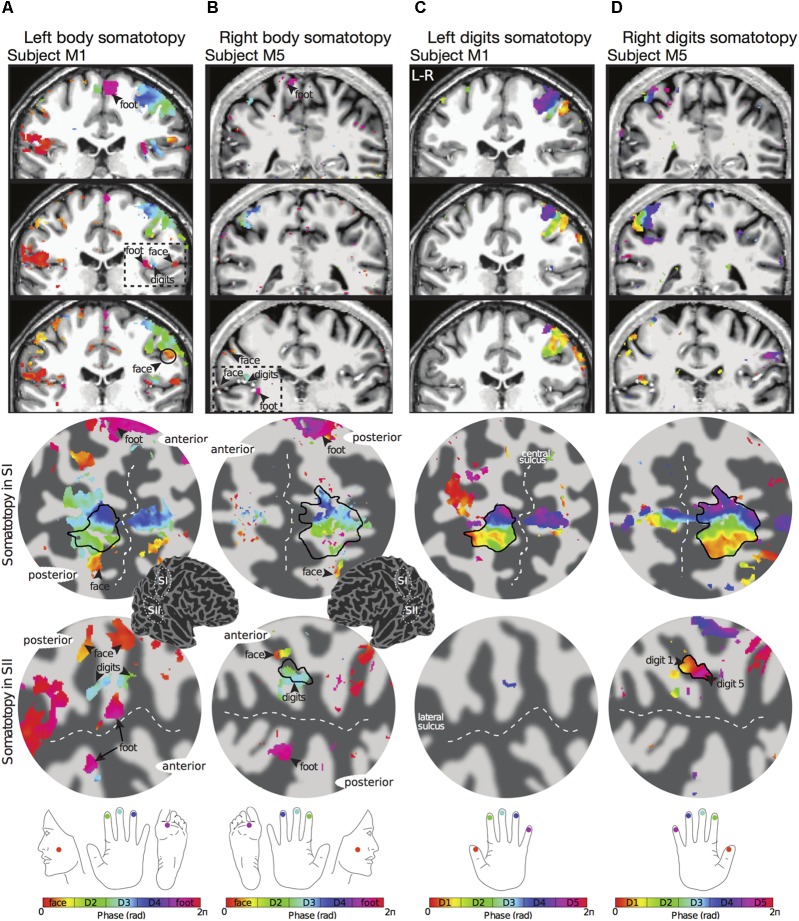
Examples of body (face–hand–foot) and digit somatotopy in two individual subjects (subjects M1 and M5). **(A)** Left face–hand–foot somatotopic (phase) map of subject M1 displayed on coronal anatomical sections (top row) and flattened cortical patch of the right central sulcus (middle row) and right lateral sulcus (bottom row). Phase values (in radians) represent preferred stimulus location (face, digit 2, digit 3, digit 4 or foot). Black arrows show the localization of the face (phase values encoded by orange) and foot (phase values encoded by magenta). The black dashed line in the anatomical space indicates the lateral sulcus, which displays a clear face–hand–foot SII somatotopy. Phase maps displayed at *c* > 0.35. Dark grey, areas of negative curvature (sulci); light grey, areas of positive curvature (gyri). The localization of the cortical patches are shown in the cortical 3D-model of the right hemisphere. **(B)** Right face–hand–foot somatotopic map for subject M5 displayed as in **(A)**. **(C)** Corresponding left hand digit somatotopic map for subject M1. Although there is a clear orderly digit somatotopy in the posterior wall of the central sulcus (SI), no somatotopy was found in the lateral sulcus (SII) for this subject. **(D)** Corresponding right hand digit somatotopic map for subject M5. This subject displays digit somatotopy in the lateral sulcus (SII).

The travelling wave ‘body mapping experiment’ revealed orderly somatotopic maps within contralateral SI in all subjects scanned, while the somatotopic organisation was not consistent across subjects in SII. Somatotopic maps within SI where consistent across subjects, and are shown for subjects M1 and M5 in **Figures 2A,B**, respectively. Within contralateral SI, the representations of Digit 2, 3, and 4 fall on the posterior wall of the central sulcus and the anterior wall of the postcentral gyrus, following an inferior to superior, and lateral to medial organisation which is consistent with the localization of these digits from the ‘digit mapping experiment’ (**Figures 2C,D**).

**FIGURE 3:**
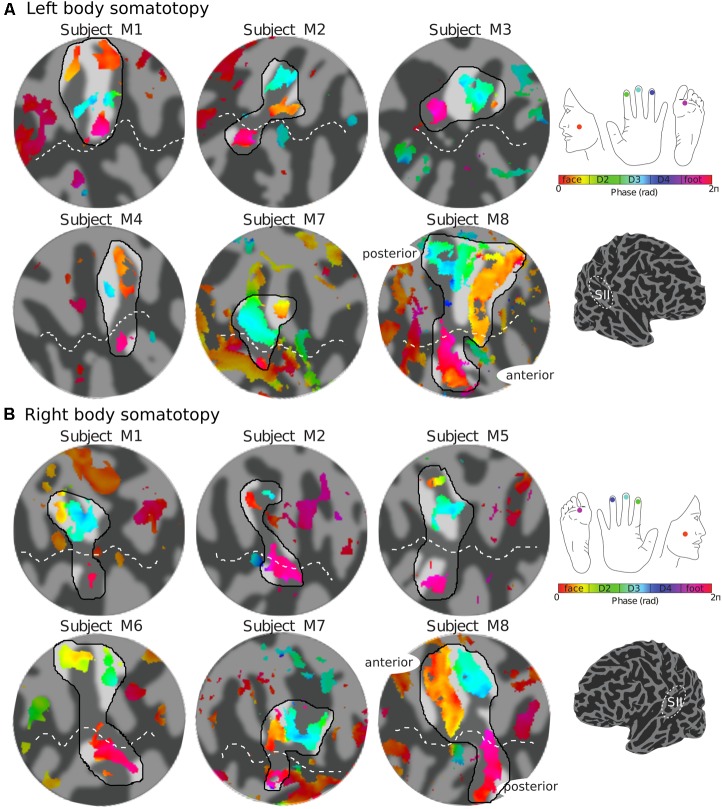
Body (face–digits–foot) somatotopy in contralateral SII for each individual subject. **(A)** Somatotopic (phase) maps of the left side of the body displayed on a flattened cortical patch of the right hemisphere. The black outlined region highlights the expected face–hand–foot axis from lateral to medial which can be observed in the group maps (**Figure 4)**. **(B)** Right side somatotopy displayed in the contralateral (left) cortex. Phase maps displayed at *c* > 0.35.

**FIGURE 4:**
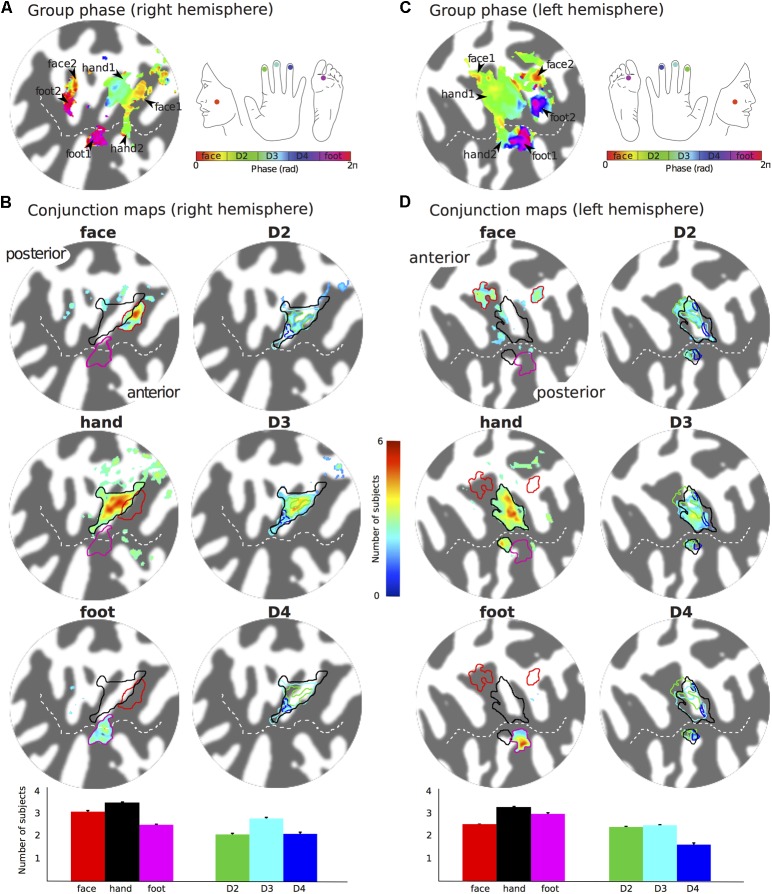
Group maps displayed on flattened patches of the right **(A,B)** and left **(C,D)** hemispheres reconstruction of the Colin27 brain template. **(A)** Group average phase map displayed at a coherence greater than 0.25. **(B)** Probability maps showing the number of subjects that activated a given location in SII for the left face, hand (any of the digits) and foot stimulation sites (left column, maps displayed at probability > 2 subjects) and the individual digits (right column, maps displayed at probability > 1.5 subjects). Bar plots show the mean number of subjects that show activation for the specific stimulation condition across the voxels in the ROI. Error bar: standard error. **(C,D)** as for **(A,B)** but for the right side somatotopy displayed on contralateral (left) cortex.

The representation of the face can be seen to be inferior to the representation of the digits in the posterior wall of the central sulcus, whilst the foot is represented in the medial wall of the anterior parietal lobe. A somatotopic arrangement can also be seen in SII within the OP, with the representation of the face (orange–yellow) located most lateral and superior, the foot (magenta) being most medial and inferior, and the representation of the digits (cyan) being between the face and foot representation. The three middle digits are not independently resolved in SII, but the phase reflects a preference for Digit 3, which was stimulated between Digits 2 and 4. Notice that for subject M1 (**Figure 2A**) there are multiple clusters for the representation of the face, digits, and foot. There was considerable variation in the number and exact location of activations for the representation of the face, digits and foot in SII across subjects (see **Figures 3A,B** for left-side and right-side stimulation, respectively). There was at least one active area for each stimulation condition in each subject (although the representation of the face was very small for subject M3). Generally, the contralateral representation of the face (orange–yellow), in both right and left hemispheres, was most anterior whilst the representation for the foot (magenta) was most medial and inferior (see activation clusters within the outlined region). The face representation was in most cases superior (and lateral) with respect to the hand, however, for subject M2 both the left and right face representations were inferior with respect to the hand, whereas for subject M7 (left hemisphere) and subject M8 (both hemispheres) the face does not appear to be neither medial nor lateral with respect to the hand representation, but it is just anterior to the hand. Some subjects showed two clusters for face representation (**Figure 3A**: subjects M1, M7, and M8; **Figure 3B**: subjects M1, M5, M6, and M7), for hand digits (**Figure 3A**: subjects M1, M2, M3, M4, M7, and M8; **Figure 3B**; subjects M2, M6, and M8) and for foot representation (**Figure 3A**: subjects M1, M2, M4, and M8; **Figure 3B**: subjects M1, M2, M5, M6, and M8). The group maps (**Figure 4**) reveal a clear organisation along the lateral sulcus in both the left and right hemispheres. For left side stimulation (right hemisphere) the group phase map (**Figure 4A**) shows an organised somatotopy with two representations for the face, hand and foot: the face (orange–yellow) representations were more superior (lateral), with the main representation (face1) most anterior and an additional representation (face2) most posterior; the main hand digits (green–cyan–blue) representation (hand1) was posterior to the representation of the face1 localization and an additional, much smaller hand representation (hand2) was located medially with respect to the face1 cluster; and there were two posterior foot (magenta) representations, one most inferior and medial (foot1) and one most posterior just inferior (foot2) with respect to the face2 localization. The probability maps (**Figure 4B**) show the same organisation, but only one cluster was revealed for the face, hand and foot using this method. These data also show that the localization of the foot was more variable across subjects, with a maximum of four subjects showing overlap in at least one vertex, in contrast with the face and hand digits where all six subjects show overlap. **Table 2** shows the variability in location of each of the activation across subjects; the mean distance across subjects ranges from 6.0 ± 8.3 mm for the hand1 localization to 12.8 ± 10.3 mm for the hand2 localization, which are less than the distances across the different pairs of activation clusters in the group phase map (mean distance of 23.5 ± 2.3 mm across the different pairs of clusters) shown in **Table 3**. When considering the probability map of the individual digits, a trend of somatotopic organisation within the hand area (black outline) also existed, though it was less well defined, with D3 taking most of the representation. However, D2 (green outline) appears to be more anterior and superior (lateral) with respect D4 (blue outline). The group phase (**Figure 4C**) and probability maps (**Figure 4D**) in the contralateral (left) hemisphere for the right side stimulation show two areas responding to stimulation of the face, the hand digits and the foot group face map only), with the main clusters for the face-hand-foot are organised from anterior to posterior and superior (lateral) to inferior (medial), mirroring the somatotopic organisation between right and left hemispheres. The additional representation for the face (face1) was located superior and more posterior to the hand representation, and the additional hand representation (hand2) was a smaller cluster located just below the first cluster, anterior with respect to the representation of the foot cluster. An additional cluster was observed for the foot representation (foot2) more posterior and superior to the first cluster in the group phase map only. The probability maps show that the representations of the face were more variable across subjects with a maximum of 4 subjects showing overlap in the face clusters compared to 6 for the digits and foot. The variability in location of the foot2 cluster was the smallest across subjects (5.0 ± 3.2 mm), with the face2 cluster having the largest (11.9 ± 7.4 mm) variation, which again are small compared with the mean distance (24.0 ± 2.2 mm) across the different activation clusters in the group phase map (see **Tables 2**, **3** for distance details). The probability maps for the individual digits also show a trend for somatotopic arrangement within both hand digit areas (black outlines) which again mirrors the organisation in the right hemisphere, with D2 (green outlines) being more anterior and superior (lateral) than D4 (blue outlines). The progression of phase values from yellow–green–cyan–blue within the hand area is also indicative of this digit somatotopic arrangement.

**Table 2:** Distance in mm between the group (phase map in **Figures 4A,B**) and each individual subject and average distance (±standard error) across subjects for the different stimulation conditions.

	Right SII					
	
	Face1	Face 2	Hand1	Hand 2	Foot1	Foot2
M1	11.6	9.2	3.6	20.4	13.3	1.3
M2	5.8	x	4.1	4.8	2.8	2.1
M3	2.6	x	0.9	29.3	12.6	x
M4	6.7	x	0.9	10.3	5.5	12.8
M7	16.1	24.5	22.8	1.5	17.1	x
M8	6.7	1.0	3.9	10.4	3.4	9.2
Average	8.3 ± 4.8	11.6 ± 11.9	6.0 ± 8.3	12.8 ± 10.3	9.1 ± 6.0	6.3 ± 5.5

	**Left SII**					

	**Face1**	**Face2**	**Hand1**	**Hand2**	**Foot1**	**Foot2**
M1	5.0	14.0	5.6	x	3.8	5.0
M2	13.0	20.7	6.6	4.3	5.9	6.5
M5	14.8	x	8.1	x	3.8	4.0
M6	1.4	8.4	6.8	17.2	3.6	9.4
M7	21.7	x	11.3	x	13.8	x
M8	15.2	2.8	9.6	11.4	4.7	4.3
Average	11.9 ± 7.4	11.5 ± 7.7	8.0 ± 2.1	11.0 ± 6.5	5.9 ± 4.0	5.0 ± 3.2


**Table 3:** Distance in mm between each pair of activation clusters in the group phase map from **Figures 4A,B** (mean and standard error across pairs of clusters also provided).

	Right SII	Left SII
Face1 – Face2	31.1	30.2
Face1 – Hand1	6.6	16.5
Face1 – Hand2	15.0	30.9
Face1 – Foot1	31.8	39.3
Face1 – Foot2	34.6	33.3
Hand1 – Face2	24.5	22.0
Hand1 – Hand2	14.7	16.3
Hand1 – Foot1	28.1	23.0
Hand1 – Foot2	28.0	18.9
Foot1 – Face2	31.2	31.1
Foot1 – Hand2	19.2	11.6
Foot1 – Foot2	23.9	18.4
Face2 – Hand2	32.9	32.6
Face2 – Foot2	10.6	13.2
Hand2 – Foot2	31.6	22.6
Mean ± SEM	23.5 ± 2.3	24.0 ± 2.2


**FIGURE 5:**
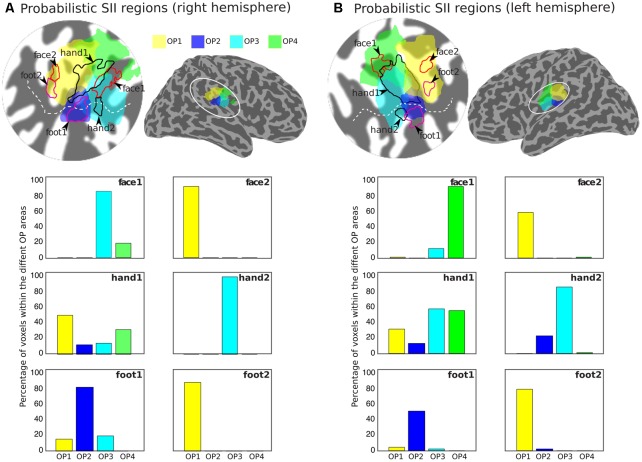
Arrangement of the group activation maps with respect to the probabilistic regions of the parietal operculum (SII) for the right **(A)** and left **(B)** hemispheres. Delineation of the face (red outlines), hand digits (black outlines), and foot (magenta outlines) activations based on the union of the group phase map (based on the binned phase data at *c* > 0.25) with the probability maps (>2 subjects) shown in **Figure 4**. Probabilistic regions as defined by the Jülich histological atlas are displayed at probability > 25%: OP1 (yellow) and OP4 (green) are more lateral and OP2 (blue) and OP3 (cyan) more medial. The bar plots show the proportion of voxels in each activation ROI that fall within each of the different probabilistic OP areas.

**Figure 5** shows the arrangement of the face (red outlines), hand digits (black outlines) and foot localization (magenta outlines), considered from the union of the definitions from the group phase and probability maps, with respect to the probabilistic cyto-architectonic OP areas; for both hemispheres the contralateral representation of the face (face1) falls close to the border between OP3 and OP4 with the additional face representations (face2) in OP1; the main representation of the digits (hand1) falls at the intersection of all OP areas and the smaller hand2 representation falls mainly within OP3 (and close to the border with OP2 for the right digits representation); one of the foot representation (foot2) falls within OP1 and the other (foot1) falls within OP2. **Figure 5** also provides for each activation area the proportion of voxels that falls within each of the OP subdivisions.

### Digit Somatotopy

**FIGURE 6:**
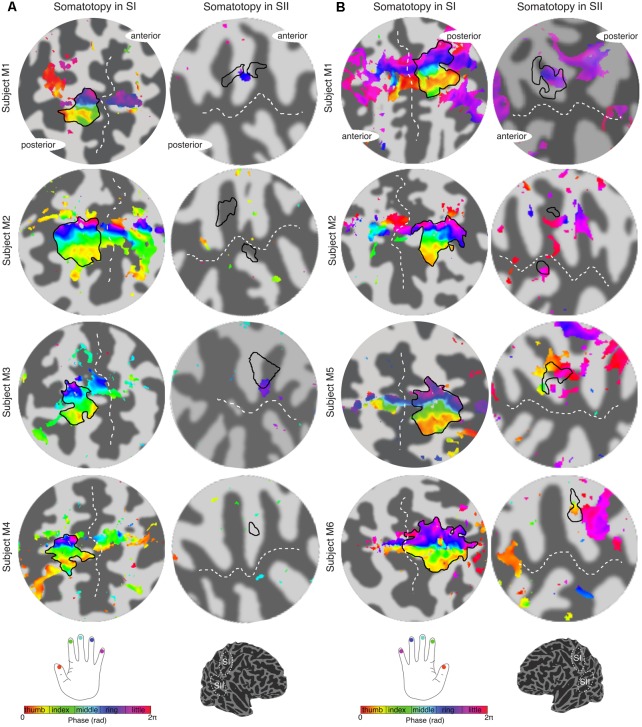
Digit somatotopy in contralateral SI and SII for each individual subject. **(A)** Left hand digit somatotopy in flattened cortical patches of right SI (left column) and SII. All subjects display a clear ordered digit somatotopy in SI (phase maps are displayed at *c* > 0.35) there is no evident somatotopy in SII (note that these phase maps are displayed at a lower *c* > 0.3). The black outlines in the SII indicate the border of the hand digit area delineated from the face–hand–foot experiment. Dashed white lines indicate the location of the central sulcus and lateral sulcus in SI and SII, respectively. **(B)** Right hand digit somatotopy displayed in contralateral cortex as in **(A)**.

The travelling wave ‘digit mapping experiment’ revealed a clear orderly pattern of phase variation in the posterior bank of the contralateral central sulcus (SI), with an inferior to superior, lateral to medial representation of Digits 1 to 5 for all subjects (**Figure 6**). In contrast, contralateral SII showed generally weak responses to individual digit stimulation, and only one subject showed a digit somatotopic pattern in SII (subject M5, **Figure 6B**). In this subject, the representation of Digit 1 (orange–yellow) appears superior and lateral with respect to Digit 5 (magenta). Note that some subjects (M1 for both left and right digit stimulation, M2 for left digit stimulation, M3, M4 and M5) present with an additional representation of Digit 2 inferior to Digit 1 in SI, which has been previously described (Besle et al., 2013, 2014).

**FIGURE 7:**
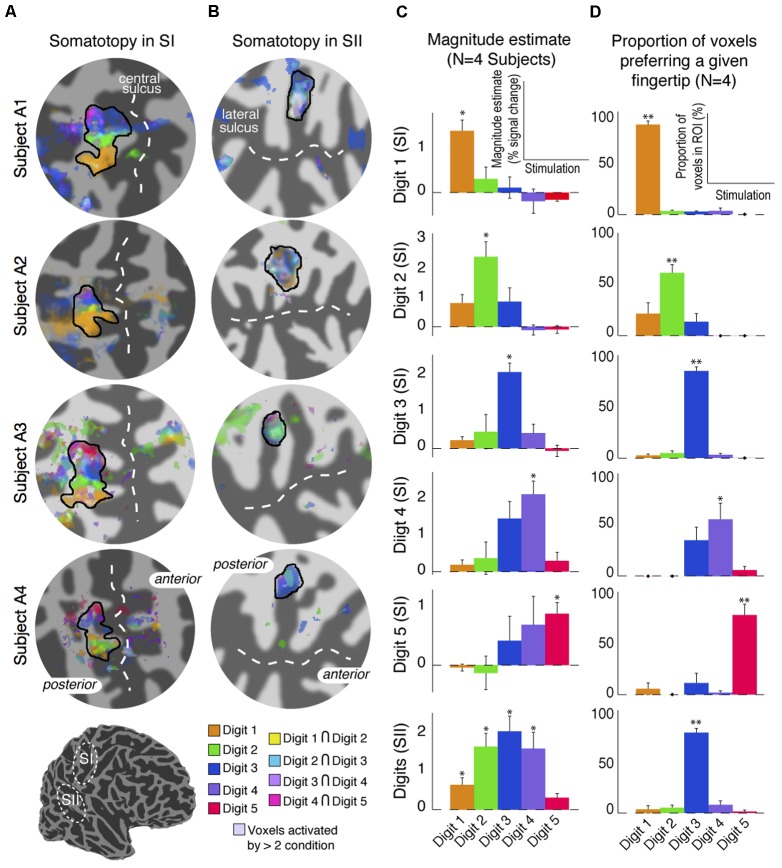
Results from the digit mapping GLM analysis. **(A)** Superposition of digit activation maps (threshold at *P* < 0.01, FDR-corrected) displayed on a flattened cortical patch of the central sulcus. Voxels activated by the stimulation of adjacent digits are shown in intermediate colours (see colour legend). **(B)** Superposition of digit activation maps on a flattened cortical patch of the lateral sulcus showing overlap of activation in SII. Voxels activated by more than two digits are shown in de-saturated (whiter) shades. **(C)** Parameter estimates of the magnitude component from the canonical HRF GLM fit for each stimulation condition averaged across voxels, and across four subjects, in each of the digit-specific SI ROIs and the SII ROI. Error bars represent standard error across subjects. ^∗^*p* < 0.05, statistical significance of each parameter estimate compared with 0. **(D)** Proportion of voxels in each of the SI digit-specific and SII ROI responding maximally in either of the five-digit stimulation conditions. Error bars represent standard error across subjects. ^∗∗^*p* < 0.01, statistical significance of each proportion compared with 0.

The digit somatotopy within SI and SII was also assessed from the ‘independent-stimulation experiment’. Stimulation of the individual digits produced robust activation patterns for each digit in the posterior bank of the central sulcus (SI), and parietal operculum (SII) for all four subjects scanned (**Figure 7**). Within contralateral SI, digit stimulation led to the expected somatotopic organisation along the mediolateral and superior to inferior axis from Digit 5 to Digit 1 (**Figure 7A**), as in the ‘digit mapping experiment’. Within SII, there was again no clear digit somatotopic pattern, with the responses to the stimulation of each digit largely overlapping, which is illustrated by the ‘whitish’ regions in Subjects A1 and A2 (**Figure 7B**). **Figure 7C** plots the average parameter magnitude estimates of the BOLD responses across voxels of the individual digit ROIs in SI for each digit stimulation condition and the contralateral SII ROI. Greater specificity (less overlap) is seen for SI compared to SII digit representations; individual digit SI ROIs are maximally activated by the stimulation of the corresponding digit, although all SI ROIs also show positive responses for adjacent digit stimulations. In contrast, SII is significantly activated by all digit stimulation conditions, although modulation by the three middle digits was larger and most voxels were maximally activated by Digit 3 (**Figure 7D**).

## Discussion

We studied the topographic organisation in SI and SII responses using fMRI with a multiband acquisition at 7T to achieve high spatial resolution within a reasonable sampling time. Compared to research in other modalities, particularly vision and audition, there is currently limited knowledge regarding the functional organisation of human somatosensory cortex. We show an orderly face–digits–foot somatotopy in contralateral SI and SII, but largely overlapping digit representations in SII.

Results from the ‘digit mapping travelling wave experiment’ revealed a clear digit somatotopic arrangement in contralateral SI, with Digits 1 to 5 mapped from lateral to medial and inferior to superior locations along the posterior wall of the central sulcus and post-central gyrus, as described in previous 7T fMRI studies (Sánchez-Panchuelo et al., 2010; Stringer et al., 2011; Besle et al., 2013, 2014; Martuzzi et al., 2014). The ‘body mapping experiment’ showed that the face SI representation is localised in the inferior part of the postcentral gyrus, inferior to the digit representations, in agreement with previous fMRI studies (Eickhoff et al., 2007; Kopietz et al., 2009), whilst the location of the foot is in the superior part of the central sulcus, as previously shown (Ruben et al., 2001; Akselrod et al., 2017).

In contrast with the clear somatotopy arrangement in SI, somatotopy in contralateral SII was less well defined and more variable (different number of clusters and localization of the different body sites) across individual subjects and hence more difficult to interpret. Despite the larger voxel size (>13 times the volume of voxels used in our study) and relative larger body site areas stimulated in a previous fMRI study, Disbrow et al. (2000) also reported great inter-subject variability in the somatotopic maps in SII, and argued that this was consistent with the variability across topographic maps in monkey observed with electrophysiology recordings (Merzenich et al., 1987). However, despite the apparent variability of the individual maps, the probability maps indicate that there is quite a large overlap of the activation clusters for each of the stimulation condition across subjects, suggesting that patterns are similar across subjects. The process of normalising to the Colin27 template brain may have reduced anatomical variabilities which also contribute to the observed inter-subject variability of the functional maps seen in **Figure 3**. The face and foot could be spatially resolved from the digits, with the representation of the face being more medial and superior to the digits and the foot being more lateral and inferior to the digits, in agreement with previous fMRI experiments (Ruben et al., 2001; Eickhoff et al., 2007), but individual digits could not be discriminated.

### Face Representation in Human Parietal Operculum

Our results show that the representation of the face was most anterior and lateral for most subjects. In three subjects there was also an additional representation for the left face stimulation and in four subjects an additional representation for the right face stimulation. When considering the group data with respect the OP, both the left and right face representations show a cluster between the borders of OP3 and OP4, rather than the OP1 and OP4 border previously reported (Eickhoff et al., 2007), although these authors also noticed that for the left representation this cluster also extended to OP3. Also consistent with (Eickhoff et al., 2007), we found an additional, more posterior cluster within OP1 for the right face representation.

### Foot Representation in Human Parietal Operculum

For both left and right foot stimulations we identified two clusters: one more medial with respect to the hand digits representations, located within OP2 near the border (and in the case of the left foot also extending to) OP3, and a second, more posterior cluster within OP1. Four/five of the six subjects scanned showed an additional representation for the left/right foot, respectively. The first cluster (OP2) could be observed in all individual subjects scanned, and this localisation is highly consistent with functional representations for the leg previously observed at the border between contralateral OP2 and OP3, but mainly in OP2 (Eickhoff et al., 2007; Bao et al., 2012). Other studies using foot stimulation with parallelepiped objects of different shape (Young et al., 2004) and vibrotactile stimulation (Beauchamp et al., 2007) also reported sparse representations of the foot in contralateral OP3 and OP2 beside OP1 and OP4. Interestingly, Beauchamp et al. (2007) reported a greater proportion of voxels activated in OP1 and OP2 than OP3 and OP4 for hand and foot stimulation.

### Hand Digit Representation in Human Parietal Operculum

For both left and right hand digits we observed a main representation medially to the activation for face stimulation, located right in the middle of the OP. This extended to OP1, OP4, OP3 and also to a lesser extent to OP2. This activation is consistent with findings by Eickhoff et al. (2007), who reported a single extended activation cluster in contralateral SII at the border between OP1 and OP4, which also extended to OP3. However, we also observed an additional, much smaller representation located more anterior and medially within OP3 for both left and right hand digit stimulation. Two representations for the left and right hand digits were seen in six/three of the six subjects for the left/right hand, respectively. In the lateral sulcus of non-human primates, three distinct anatomical areas (SII, PV, and VS) have been defined (Burton et al., 1995; Krubitzer et al., 1995), each of these with a complete somatotopic map of the whole body. In this organisation, SII and PV maps constitute mirror images of each other, bordering at the representations of the face (lateral), hands (intermediate), and feet (medial), with shoulder, trunk, and legs represented further apart from this border in the anterior part of PV and the posterior aspect of SII. The VS subdivision, medial to SII and PV, contains a crude somatotopic map in which the head is represented most anteriorly. Disbrow et al. (2000) were the first to observe, using fMRI, that human SII may contain several somatotopically organised areas homologous to non-human primates. Eickhoff et al. (2007) later concluded that OP1, OP4 and OP3 may constitute the human homologues of areas SII, PV, and VS, respectively. Our findings are consistent with these previous fMRI studies which found one single cluster at the border of OP1/OP4, however, in contrast to our study, which only stimulated sites represented at the border between OP1/OP4, these studies also stimulated the trunk, which allowed them to demonstrate the double somatotopy within the OP. The additional representation we found within OP3 is also consistent with the existence of a third somatotopic map described as the human homologue of monkey VS.

Despite the much higher spatial resolution of this study, 1.5 mm isotropic voxels compared to a previous study with 4 mm isotropic voxels (Ruben et al., 2001), individual contralateral digits in SII could not be resolved spatially in our maps. In SII, the digit mapping travelling wave paradigm did not reveal any significant activation to any of the digits, except for in one subject (**Figure 2D**), where there was phase variation between Digits 1 and 5. This organisation with Digit 1 being located more anterior and superior (and lateral) with respect to Digit 5 is consistent with the digit organisation described in the electrophysiology of non-human primates (Krubitzer et al., 1995). The lack of a digit somatotopic map in SII suggests that the representation of the individual fingers in SII largely overlap, in line with previous neurophysiological studies performed in macaque monkeys (see Taoka et al., 2016) reporting complex somatosensory responses in SII compared to SI, with large receptive fields covering multiple body parts (Robinson and Burton, 1980; Sinclair and Burton, 1993). The travelling wave paradigm locates regions of the brain which preferentially respond to stimulation of a given digit, hence this paradigm is less effective at identifying neural populations with wide receptive fields which overlap across multiple digits (Besle et al., 2014). Results from the ‘independent stimulation’ digit mapping paradigm, for which each digit is modelled as an on–off block design, indeed suggest that the representation of the digits largely overlap (**Figure 7B**), supporting this statement. A potential limitation of these data is the differential effect of attention on the individual digits; the increased response to Digits 2, 3, and 4 compared to Digits 1 and Digit 5 in SII (**Figure 7C**) is possibly due to the task requiring subjects to pay greater attention to Digits 2, 3, or 4 than Digits 1 or 5. However, for this task, spatial digit specificity in SI was observed for the cortical representation of all digits and the responses for digit-specific regions of SI were maximal for the corresponding digit, including Digits 1 and 5, despite subjects only attending to Digits 2, 3, or 4. Hence, this potential attentional confound does not invalidate the conclusion that responses are more spatially digit specific in SI than in SII.

## Conclusion

The use of multiband acquisition at 7T has allowed the simultaneous assessment of somatotopic mapping of SI and SII with higher spatial resolution (1.5 mm isotropic) than has previously been achieved. Despite the existence of a clear digit map within SI, the representation of individual fingers within SII cannot be resolved in individual subjects, although a trend for digit somatotopy within the hand area was revealed in the group probability maps. In contrast, clear separation of body areas of the face–hand–foot is revealed in SII.

## Author Contributions

All authors contributed to the conception and design of the work. RSP and SF developed the imaging protocols. RSP, SF, and MH acquired the data. RSP and JB analysed the data. RSP drafted the original manuscript. All authors contributed to the interpretation of the results and revisions of the paper.

## Conflict of Interest Statement

The authors declare that the research was conducted in the absence of any commercial or financial relationships that could be construed as a potential conflict of interest.

## Footnotes

1 www.dancerdesign.co.uk

2 https://github.com/justingardner/mrTools

3 http://www.cns.nyu.edu/heegerlab

4 http://surfer.nmr.mgh.harvard.edu/

5 http://white.stanford.edu/software/

6 http://www.bic.mni.mcgill.ca/ServicesAtlases/Colin27
